# Untangle the Multi-Facet Functions of Auts2 as an Entry Point to Understand Neurodevelopmental Disorders

**DOI:** 10.3389/fpsyt.2021.580433

**Published:** 2021-04-23

**Authors:** Wenbin Pang, Xinan Yi, Ling Li, Liyan Liu, Wei Xiang, Le Xiao

**Affiliations:** ^1^Key Laboratory of Brain Science Research & Transformation in Tropical Environment of Hainan Province, Hainan Medical University, Haikou, China; ^2^National Health Commission (NHC) Key Laboratory of Control of Tropical Diseases, Hainan Medical University, Haikou, China; ^3^Department of Pediatric Rehabilitation, Hainan Women and Children's Medical Center, Haikou, China

**Keywords:** autsim spectrum disorder, transcriptional regulation, epigenetic regulation, synapse formation, Auts2

## Abstract

Neurodevelopmental disorders are psychiatric diseases that are usually first diagnosed in infancy, childhood and adolescence. Autism spectrum disorder (ASD) is a neurodevelopmental disorder, characterized by core symptoms including impaired social communication, cognitive rigidity and repetitive behavior, accompanied by a wide range of comorbidities such as intellectual disability (ID) and dysmorphisms. While the cause remains largely unknown, genetic, epigenetic, and environmental factors are believed to contribute toward the onset of the disease. Autism Susceptibility Candidate 2 (Auts2) is a gene highly associated with ID and ASD. Therefore, understanding the function of Auts2 gene can provide a unique entry point to untangle the complex neuronal phenotypes of neurodevelpmental disorders. In this review, we discuss the recent discoveries regarding the molecular and cellular functions of Auts2. Auts2 was shown to be a key-regulator of transcriptional network and a mediator of epigenetic regulation in neurodevelopment, the latter potentially providing a link for the neuronal changes of ASD upon environmental risk-factor exposure. In addition, Auts2 could synchronize the balance between excitation and inhibition through regulating the number of excitatory synapses. Cytoplasmic Auts2 could join the fine-tuning of actin dynamics during neuronal migration and neuritogenesis. Furthermore, Auts2 was expressed in developing mouse and human brain regions such as the frontal cortex, dorsal thalamus, and hippocampus, which have been implicated in the impaired cognitive and social function of ASD. Taken together, a comprehensive understanding of Auts2 functions can give deep insights into the cause of the heterogenous manifestation of neurodevelopmental disorders such as ASD.

## Introduction

Neurodevelopmental disorders are a group of diseases characterized by deficits in the functions of the brain that affect a child's behavior, memory, or ability to learn, including intellectual disability (ID), autism spectrum disorder (ASD), attention-deficit hyperactivity disorder (ADHD) and so on (1). The symptoms of these disorders typically start during early childhood, when the nervous system undergoes intensive development and maturation, indicating that perturbed neurodevelopment contributes to the onset of diseases (2). In a patient, more than one neurodevelopmental disorder often appears as comorbidities, suggesting common neurological etiology shared by them (3). ASD is one of neurodevelopmental disorders, affecting ~1% of the population (4). The patients often showed impaired social communications, restricted interests and repetitive behaviors (5, 6). Although, patients with autism usually have these core symptoms in different degree, they also display a wide range of comorbidities, including ID, ADHD, motor control deficit, epilepsy, anxiety, and sleep disorders (3). Moreover, ~20% of the patients show dysmorphic features, varying from hypertelorism, strabismus, microcephaly and/or macrocephaly (7, 8).

Despite the wide spectrum of phenotypes observed in patients, the etiology of ASD remains largely unclear. It was proposed that the clinical manifestations of ASD patients are the sum-up effect of genetic, environmental factors, and gene-environment interactions (9). According to the summary of AutismKB, ~3,000 genes and ~4,900 copy-number variants were associated with the risk of ASD. Among them, 171 autism-related genes were considered to have high confidence for the causality link (http://autismkb.cbi.pku.edu.cn). The proteins encoded by the high-risk genes include synaptic receptors and cell adhesion molecules such as NRXN, scaffolding proteins and the actin cytoskeleton such as SHANKs, as well as transcription factor such as *Auts2* (10). However, the autism patients in which a genetic cause could be identified only account for ~25% of the total number of cases. The etiology of vast majority (~75%) cases remain undefined (11).

Recent studies have shown that epigenetic regulations such as, DNA methylation and histone modification are associated with ASD (12, 13). Furthermore, prenatal exposure to chemicals such as endocrine-disrupting compounds (EDCs) or maternal stress have been linked to higher risk of neurological and psychiatric disorders in the offspring including ASD (14, 15).

Due to the heterogeneity of the phenotypes displayed in ASD, the complexity of genetics and epigenetic risk factors, as well as the mostly unknown pathophysiological mechanisms of the disease, there is still lack of effective pharmacological treatment for ASD. Intriguingly, Balovaptan, a selective small molecule antagonist of the vasopressin receptor showed specific effect in improving social interaction and communication of ASD male patients with normal IQ level, according to its phase II clinical trial (16). Therefore, to understand the function of a single ASD risk-gene remains to be an important entry point in order to untangle the complexed neurological mechanisms during the onset and progressing of ASD.

**Figure 1:**
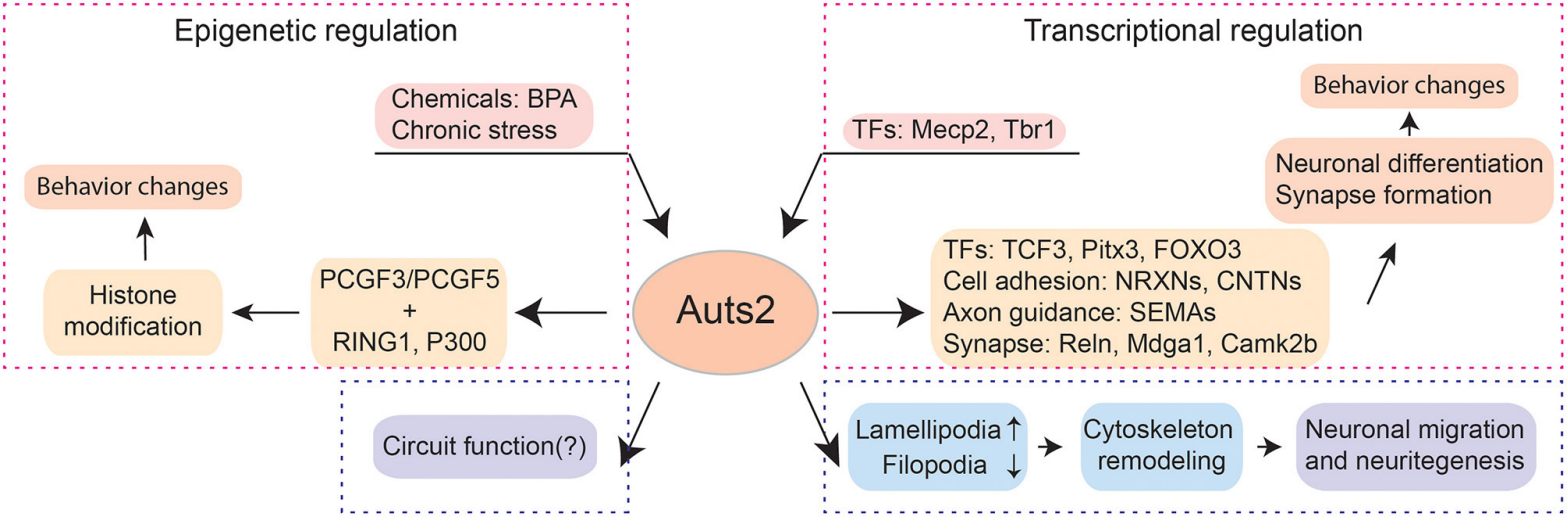
The multi-facet functions of Auts2. Auts2 participates in transcriptional and epigenetic regulations, neuronal differentiation, neuronal migration, neuritegenesis as well as synapse formation. In the aspect of transcriptional regulation, the expression of Auts2 is controlled by transcriptional factors (TFs) such as Mecp2 and Tbr1. Moreover, Auts2 is a transcriptional regulator itself. It can control the expression of other TFs (e.g., TCF3, Pitx3, and FOXO3), cell adhesion molecules (e.g., NRXNs and CNTNs), axonal guidance molecules (e.g., SEMAs) and proteins involved in synaptic functions (e.g., Reln, Mdga1, and Camk2b). The precise coordinate of transcription by Auts2 is critical for neuronal differentiation and synapse formation. The deletion of Auts2 gene can lead to behavior impairment in social interaction, cognition, and ultrasonic vocalization, which captures the features observed in patients with neurodevelopmental disorders such as ASD. Auts2 also plays important roles in epigenetic regulations. After binding PCGF3/PCGF5, RING1 and P300, the Auts2-containing complex can bind to the promotor regions of various genes and modify histones. Through epigenetic regulation, Auts2 can mediate the effect of environmental factors, e.g., chemicals and maternal health factors on the nervous system. Cytoplasmic Auts2 regulates the neuronal migration and neuritegenesis through inducing lamellipodia and suppressing filopodia. The function of Auts2 in circuits remains to be further explored.

Autism Susceptibility Candidate 2 (Auts2, also named as the “activator of transcription and developmental regulator” by the HUGO gene Nomenclature Committee (HGNC), #14262) was first identified as the genetical cause ASD in a pair of monozygotic twins (17). In addition, the chromosome structural rearrangement or copy-number variance of the Auts2 locus has been associated with a wide range of neurological and psychiatric conditions such as ID, epilepsy, bipolar disorders, alcohol abuse, ADHD, and schizophrenia (18–23). Notably, 80~100% patients with Auts2 disruption were diagnosed with ID or developmental delay, and ~40% patients had ASD (18, 24–26). Some patients displayed dysmorphic features such as short stature, microcephaly, and facial changes (18, 26, 27). Thus, the complexity of phenotypes caused by Auts2 mutations or genetic variations are consistent with the broad range of symptoms manifested by neurodevelopmental disorders, indicating its comprehensive roles in the development of brain functions (Figure 1, Table 1).

**Table 1:** Summary of key findings about the multi-facet functions of Auts2.

**Domains**	**Key findings about the functions of Auts2**	**References**
Transcriptional regulation	Long isofroms of Auts2 delayed neuronal differentiation in mouse embryonic stem cells.	(28)
	Auts2 bound to regulatory elements of Pitx3, TCF3, FOXO3, NRXN1, CNTN4, RBFOX1, and ATP2B2.	(29)
	In Auts2 knock-out mice, genes such as, Reln, Mdga1, Camk2b, Cacnalc and C1ql-family were differentiately expressed.	(30)
	The transcription of Auts2 was regulated by Mecp2 and Tbr1.	(31–34)
Epigenetic regulation	The short isoforms of Auts2 bound to two polychomb proteins, PCGF3, and PCGF5.	(28, 35)
	Auts2-containing PRC1 suppressed gene expression, but after recruiting P300, it became a transcriptional activator.	(28, 35)
	Auts2-containing PRCs modified histone H3 acetylated at lysine 27 and trimethylation at lysine 4.	(29, 35)
	Auts2 activated the transcription of ganglioside-producing enzyme.	(36)
	Prenatal exposure to BPA decreased expression of Auts2 in neonatal mice.	(37)
	Maternal thermal stress in zebrafish induced down-regulation of Auts2 in the eggs.	(38)
	Chronic stress induced a DNA adenine modification at Auts2 and its regulator Tbr1.	(39)
Synapse formation	Auts2 controlled the number of excitatory synapses and synchronized the balance between excitation and inhibition in the brain.	(30)
Cytoskeleton regulation	Auts2 induced lamellipodia *via* activation of Rac1 and suppressed filopodia *via* down-regulations of Cdc42.	(40)
	Auts2 knock-out mice showed impairment in both migration and axon elongation of cortical neurons.	
Regions expressed	The expression of Auts2 in the brain was strong by E16, then decrease to a low level after P21.	(34, 40)
	In cerebral cortex, the expression of Auts2 exhibited a strong rostral-caudal gradient.	
	In hippocampus, Auts2 was expressed in the denta gyrus, CA1 and CA3 from E14 onwards, and Auts2 was located to granule cell layer and subgranular zone by P21.	
	In cerebellum, Auts2 was in granule neurons, precursor of Purkinje cells and deep nuclei at early developmental stage. By P21, Auts2 was expressed in Purkinje cells only.	(34)
	On E14, Auts2 was expressed in dorsal thalamus. By P21, Auts2 was in ATN, and VL/VM.	

Auts2 gene is located on chromosome 7q11, span 1.2 Mb. It has 19 exons, coding for a protein with 1,259 amino acids in humans. The sequence is 62% conserved between zebrafish and human, and 93% between mouse and human. Sequence analysis showed that the Auts2 protein has a PY (Pro-Tyr) motif, eight CAC (His) repeats, two proline-rich domain, several putative SH2, and SH3 domains, kinase phosphorylation sites, as well as N-glycosylation sites (17, 41). Similar PY domain is also present in several transcription factors. The CAC repeats could be signals for locating at a subnuclear compartment, which associated with RNA splicing machinery (42). Therefore, the sequence of Auts2 indicated its role in transcriptional regulation. The multiple SH2 and SH3 sites and the proline-rich domain could be potential protein-protein interaction regions (41, 43).

## Auts2 is a Key-Regulator of the Complex Transcriptional Network During Brain Development

Auts2 gene has two transcription start sites, conserved in both mouse and human. One is in front of exon 1, and the other one is in exon 9, resulting in a full-length transcript (~140kDa) and short isoforms (18, 28, 40). Additional alternative splicing exons include exon 10 and 12 in human, corresponding to exon 11 and 13 in mouse. All of them were found in the developing brain, but only two of the variants were found in adult human and mouse brain (28). In mouse embryonic stem cells (mESCs), the expression of short isoform becomes dominant when neurons start to differentiate, in replacement of the long isoforms. Overexpression of the long isoforms *in vitro* resulted in delayed neuronal differentiation, and mutations in the sequence specific to the long isoforms led to accelerated differentiation. The short isoform was also more expressed than the long one in embryonic human brain (28). In developing mouse brain, the long and short isoforms are expressed in cerebral cortex at embryonic stages, but the expression of short isoforms continuously decrease since E14.5, and is barely detectable 7 days after birth. In juvenile mouse cerebral cortex, only the full-length Auts2 transcripts were detected (40). Therefore, the temporal expression pattern of Auts2 isoforms is tightly regulated, indicating its multi-facet functions during neurodevelopment. But how exactly the expression of Auts2 isoforms is orchestrated need to be further investigated.

Interestingly, Auts2 has one of the highest intronic RNA level in fetal brain (44). Moreover, mutations in the non-coding region of Auts2 gene in both zebrafish and human were associated with developmental deficit (45–47). Candidates for the enhancers of Auts2 have been mapped to several introns, and showed specific expression in embryonic fish and mouse brain (45). Studies have shown that the intron retention is an important slicing program for transcriptional regulation in neurons, and it is modulated by neuronal activity (48, 49). Thus, the abundance of intronic Auts2 RNA suggests that Auts2 might be actively participating in, as well as subjected to various transcriptional regulation during development.

The function of different isoforms can be executed through the interaction with diverse partners during transcriptional regulation, as well as other cellular process. The short isoform of Auts2 was found to be exclusively expressed in the nucleus (28, 40). Moreover, Auts2 was found to localize at the promotors and non-promotor regulatory elements related to neurodevelopment, including transcription, splicing of RNAs, proliferation of cells and migration of neural precursor cells. Auts2-associated regions also included binding motifs of transcription factors involved in neurodevelopment, such as, paired-like homeodomain 3 (Pitx3), transcription factor 3 (TCF3) and forkhead box O3 (FOXO3). In addition, Auts2 was found to occupy functionally active enhancers of neurexin 1 (NRXN1), contactin 4 (CNTN4), RNA-binding protein fox-1 homolog (RBFOX1) and ATPase Ca++ transporting plasma membrane 2 (ATP2B2) (29). A genome-wide association study (GWAS) has shown that Auts2 is a regulatory element for semaphorin 5A (SEMA5A), an axon-guidance molecule (50). Both NRXN1 and SEMA5A belong to the core ASD-risk genes (51). Selective removal of Auts2 in excitatory neurons leads to the down-regulation of NRXNs and SEMAs in hippocampi (30). In addition, many genes coding for proteins implicated in synaptic functions, e.g., Reln, Mdga1, Camk2b, Cacna1c, and C1ql-family genes are differentially expressed (30). Therefore, it is possible that Auts2 regulate synaptogenesis through modulating the transcription of relevant genes.

The transcription of Auts2 is regulated by several genes indicated in ASD. For example, methyl CpG binding protein 2 (Mecp2), a gene implicated in Rett-syndrome and ASD, represses the expression of Auts2 in both mouse and human brain (31, 32). T-Box Brain Protein 1 (Tbr1), a transcription factor that assign regional and laminar identity of postmitotic neurons, can activate the expression of Auts2 (33, 34). Thus, Auts2 is a key-regulator of the complex transcriptional network during brain development. Indeed, perturbing Auts2 expression in both zebrafish and mouse brain can both lead to various neurodevelopmental phenotypes. The zebrafish Auts2 morphants show microcephaly and smaller-jaw size, similarly to the observations from human patients (17, 29). Auts2 knock-out mice also showed a general developmental delay, including lower body weight and impaired motor skills (30, 35, 52).

## Nuclear Auts2 is a Mediator of the Epigenetic Regulation During Neurodevelopment

Auts2 was found to regulate neuronal differentiation through histone modifications, an epigenetic mechanism. The short isoform of Auts2 can bind to two polycomb proteins, PCGF3, and PCGF5, and be part of polycomb-repressive complex 1 (PRC1) for suppressing gene expression (28, 35). After recruiting P300, the Auts2-containing PRC1 were converted into a transcriptional activator from a repressor (28, 35). The histone modifications by Auts2-containing PRCs include histone H3 acetylated at lysine 27 (H3K27ac) and trimethylation at lysine 4 (H3K4me3) (29, 35). This can be one of the mechanisms that controls the metabolic reprogramming during the differentiation from stem cells towards the neural lineages. In neuronal stem-cells differentiation period, glycosphingolipid production was switched from a globo-series to ganglio-series (53). Auts2 was found to activate the transcription of rate-limiting ganglioside-producing enzyme, and thereby control the glycosphingolipid reprogramming (36). This study linked the Auts2-mediated epigenetic regulation with critical lipid metabolism during neuronal differentiations. Many questions remain to be elucidated. For example, what is the exact role of this metabolic glycosphingolipid switch for neuronal differentiation *in vivo*, as well as how this could contribute to the etiology of ASDs caused by Auts2-mutation?

Environmental chemicals and maternal health factors have been associated with increased risk of ASD (54). Exposure to ambient pesticide during prenatal period and infancy elevates the occurrence rate of Autism (55). Maternal use of selective serotonin reuptake inhibitors (SSRIs) as a result of diagnosed depression also increase the risk of ASD (56). The epigenetic regulation participated by Auts2 might be associated with environmental chemicals and maternal stress during pregnancy. Prenatal exposure to bisphenol A (BPA) lead to decreased expression of Auts2 in the hippocampi from neonatal male mice (37). In zebrafish, maternal thermal stress induced down-regulation of Auts2 in the eggs, as well as fear-related locomotor responses to a novel environment in the offspring (38). In mouse prefrontal cortex, chronic stress induced a DNA adenine modification at genes including Auts2 and its regulator Tbr1 (39), which led to a comprehensive gene expression changes in the brain and thereby might be the cause of neuropsychiatric disorders. However, there is no direct evidence so far to show that Auts2 mediated neuronal dysfunction induced by environmental factors. It is highly interesting to investigate the role of Auts2 as an epigenetic linkage for the etiology of ASD upon environmental risk-factor exposure.

## Auts2 Regulates the Numbers of Excitatory Synapses and Thus Synchronizes the Balance Between Excitation and Inhibition

The loss of Auts2 was shown to result in excessive excitatory synapses *in vitro* and *in vivo* (30). When Auts2 was selectively deleted in hippocampal neurons postnatally, the dendritic spine formation increased significantly, and more excitatory synapses were formed onto these spines. The number of inhibitory synapses were unchanged. Therefore, postnatal expression of Auts2 in the brain can modulate the balance between excitation and inhibition by limiting the number of excitatory synapses. Interestingly, the phenotype of excessive excitation in Auts2 knockout neurons could be rescued by re-expression of full-length transcripts. It could neither be rescued by the C-terminal Auts2 short isoforms, nor full-length transcripts tagged with the nuclear export sequence (30). This indicated that the function of balancing synaptic inputs by Auts2 depends on its genetic and/or epigenetic regulation onto the expression of relevant genes. The authors proposed some candidate genes such as, Reln, Mdga1, Camk2b, and Cacna1c (30), but the exact pathway through which Auts2 controls the formation of excitatory synapses remains to be further explored.

The balance of excitation and inhibition (E/I balance) within neural circuits have been proposed as one of the causes of autistic behaviors (57, 58). Increased excitation in the mouse medial prefrontal cortex (mPFC) leads to great impairment in their social behaviors (59). In mice lacking contacting associated protein-like 2 (CNTNAP2), a gene strongly associated with autism, reduced excitation, or elevated inhibition in mPFC could rescue their deficits in social behaviors (60). Besides impaired social communication, ASD patients often manifest sensory abnormality (1), which may also be due to the imbalanced E/I in sensory cortices. The mice carried specific deletion of shank3 in parvalbumin interneurons of primary somatosensory cortex exhibited higher sensitivity in relevant behavior task (61). In mice lacking of Auts2, the social interactions reduced, and the sensitivity for startle response as well as nociceptive response increased (30). Whether re-expression of Auts2 in a circuit-specific manner could rescue the social deficit and hypersensitivity remained to be answered.

## Cytoplasmic Auts2 Can Directly Regulate Cytoskeleton During Neuronal Migration and Neuritegenesis

In addition to transcriptional regulation, Auts2 is present in cytoplasm and can regulate cytoskeleton through Rho family GTPases. It induces lamellipodia *via* activation of Rac1 and suppresses filopodia *via* down-regulations of Cdc42. Genetic ablation of Auts2 *in vivo* impairs both migration and axon elongation of cortical neurons (40). These observations revealed a novel function of Auts2 in neuronal migration and neurite outgrowth. The correct assembly of cortical structure is essential for the establishment of microcircuits, and the execution of sensory, motor, social, and cognitive functions (62, 63). During development, nuclear Auts2 can modify transcription according to genetic and epigenetic cues, whereas, cytoplasmic Auts2 join the fine-tuning of actin dynamics in neuronal processes. Thus, the multi-tasking of Auts2 in early development may attribute to the complexity of phenotypes upon Auts2 mutations.

## Auts2 is Expressed in Brain Regions Related to Cognitive Functions

In developing mouse brain, Auts2 mRNA is highly expressed in the frontal cortex, olfactory bulb, hippocampus, dorsal thalamus, inferior colliculus, and substantia nigra. The expression of Auts2 mRNA in the brain was very strong by E16, then decrease to a low level after postnatal day 21 (34, 40). In cerebral cortex, the expression of Auts2 exhibits a strong rostral-caudal gradient, with a much higher expression in the frontal areas, suggesting its role in assigning regional identity of the cortex (33, 34). In hippocampus, Auts2 is expressed in the denta gyrus, CA1, and CA3 from E14 onwards, and the expression is located to granule cell layer and subgranular zone by P21, indicating a potential neurogenesis role (34). In cerebellum, the expression of Auts2 was in granule neurons, precursor of Purkinje cells, and deep nuclei at early developmental stage. By P21, it is expressed in Purkinje cells only. On E14, Auts2 was expressed in dorsal thalamus. By P21, the thalamic expression is restricted in the anterior thalamic nuclei (ATN), and in ventrolateral/ventromedial nuclei (VL/VM) (34). In human fetal brain, Auts2 was found in frontal, parietal and temporal lobes of the neocortex, as well as the ganglionic eminence, caudate nucleus, putamen nuclei, and cerebellum (41, 64).

From circuit level, the frontal cortex, hippocampus, and cerebellum have all been implicated in the impaired cognitive function of ASD (65–67). ATN and VL/VM thalamic nuclei have been found to form dense reciprocal connections with the frontal part of the cortex, particularly with the prefrontal cortex, prelimbic cortex, and anterior cingulate cortex (68, 69). Cerebellar nuclei can forward information to mPFC through VM, as well as to striatum through intralaminar thalamic nuclei (67, 70). These pathways provide rich regulations onto the brain areas that play central role in executing cognitive and social behaviors (67, 71, 72). For example, the deep layer mPFC neurons which project to the subcortical areas were recruited during social exploration. Altering the activity of these neurons could modulate the social performance in ASD mouse models (73). Moreover, activation of the Purkinje cells in crus1 could alleviate the social deficit and repetitive behaviors through modulating a multi-synaptic circuit targeted to mPFC (70). Auts2 mutant mice showed decreased social interaction as well as vocal communication (30, 35) [but see (52)]. How exactly Auts2 could implement its function upon neural circuits in these regions are still unknown. A detailed mapping of Auts2 function in a circuit-specific manner would greatly increase our knowledge for the behavioral abnormalities of neurodevelopmental disorders, and maybe lead to a novel treatment strategy.

## Conclusions and Perspective

One of the main challenges in the field of neurodevelopmental disorders is to precisely link the genetic cause with the pathophysiological changes of the nervous system. To understand the function of each gene that associated with neurodevelopmental disorders is a unique entry point to elucidate the progress of the diseases. Patients with Auts2 mutation or variations showed a broad range of symptoms, including ID and ASD, consistent with the heterogeneity of neurodevelopmental disorders. Auts2 is a key-regulator of the transcriptional network during brain development. It can control the expression of transcription factors, cell adhesion, and axon guidance molecules, as well as proteins important for synaptic functions. Moreover, through binding Polycomb proteins and forming functional complex, Auts2 participates in the epigenetic modulations, which may mediate the effect of environmental or maternal factors on brain development. In addition, Auts2 regulates the neuronal migration and neuritegenesis by cytoskeleton remodeling. Furthermore, Auts2 controls the number of excitatory synapses to achieve precise E/I balance in the central nervous system (Figure 1). A few key questions remain to be answered. For example, how the temporal expression of different Auts2 isoforms is orchestrated remains unknown. The downstream molecular pathways through which Auts2 regulates the synapse formation need to be elucidated. How exactly Auts2 exerts its influence onto cognitive and social behaviors should be best addressed in a circuit-specific manner. Thus, a comprehensive understanding of Auts2 function will help us to search for treatment strategies of neurodevelopmental disorders.

## Author Contributions

LX and WX designed the study. LX, WX, and PW wrote the paper. XY, LLi, and LLiu provided insightful discussion for the manuscript. All authors contributed to the article and approved the submitted version.

## Conflict of Interest

The authors declare that the research was conducted in the absence of any commercial or financial relationships that could be construed as a potential conflict of interest.
